# Biology and pathology of the uterine microenvironment and its natural killer cells

**DOI:** 10.1038/s41423-021-00739-z

**Published:** 2021-08-23

**Authors:** Fuyan Wang, Anita Ellen Qualls, Laia Marques-Fernandez, Francesco Colucci

**Affiliations:** 1 Department of Obstetrics & Gynaecology, University of Cambridge, National Institute for Health Research Cambridge Biomedical Research Centre, Cambridge, CB2 0SW UK; 2 Department of Biochemistry and Molecular Biology, and Zhejiang Key Laboratory of Pathophysiology, School of Medicine, Ningbo University, Ningbo, 315211 China; 3 Centre for Trophoblast Research, University of Cambridge, Cambridge, CB2 3EG UK

**Keywords:** Natural killer cells, Uterine microenvironment, Pregnancy, Decidua, uNK, Immunology, Cell biology

## Abstract

Tissues are the new frontier of discoveries in immunology. Cells of the immune system are an integral part of tissue physiology and immunity. Determining how immune cells inhabit, housekeep, and defend gut, lung, brain, liver, uterus, and other organs helps revealing the intimate details of tissue physiology and may offer new therapeutic targets to treat pathologies. The uterine microenvironment modulates the development and function of innate lymphoid cells [ILC, largely represented by natural killer (NK) cells], macrophages, T cells, and dendritic cells. These immune cells, in turn, contribute to tissue homeostasis. Regulated by ovarian hormones, the human uterine mucosa (endometrium) undergoes ~400 monthly cycles of breakdown and regeneration from menarche to menopause, with its fibroblasts, glands, blood vessels, and immune cells remodeling the tissue into the transient decidua. Even more transformative changes occur upon blastocyst implantation. Before the placenta is formed, the endometrial glands feed the embryo by histiotrophic nutrition while the uterine spiral arteries are stripped of their endothelial layer and smooth muscle actin. This arterial remodeling is carried out by invading fetal trophoblast and maternal immune cells, chiefly uterine NK (uNK) cells, which also assist fetal growth. The transformed arteries no longer respond to maternal stimuli and meet the increasing demands of the growing fetus. This review focuses on how the everchanging uterine microenvironment affects uNK cells and how uNK cells regulate homeostasis of the decidua, placenta development, and fetal growth. Determining these pathways will help understand the causes of major pregnancy complications.

## Introduction

### The decidua in humans and mice

The decidua is the specialized uterine mucosa that forms from the endometrium under the influence of progesterone at the end of each cycle in humans. The word decidua comes from the Latin *deciduous*, meaning falling down or falling off. Indeed, the decidua is shed after every menstrual cycle and after birth, when the placenta is expelled (the placenta is also known as the ‘afterbirth’). The cellular composition of the decidua includes stromal cells transformed from fibroblasts, glands, and immune cells, mostly cells of innate immunity, including ILCs (NK cells being the most abundant) and macrophages, but also some cells of adaptive immunity, including T, regulatory T (Tregs), and dendritic cells (DCs). Innate immune cells populate also the mouse decidua, which forms only in response to implantation and has a broadly similar cellular composition in both species [1–3]. The decidua is essential for healthy implantation and for the formation of the placenta [4] as illustrated by ectopic pregnancies and placenta creta or accreta [5]. Fetal trophoblast cells erode and invade the decidua in both primates and rodents. In humans, fetal trophoblast cells migrate through the decidua and up into the muscle layer, known as myometrium. While the invasion is shallower in mice, the type of placenta is haemochorial in both species—and indeed in all primates and rodents—meaning that the outermost fetal membrane called chorion bathes into the maternal blood. This makes human and mouse placentas more similar to each other than to those of other mammals such as ruminants, horses, and pigs that have epitheliochorial placentas, in which the fetal chorion remains separated by endometrial endothelium, connective tissue, and uterine endothelium from the maternal blood [6]. During decidualization and until the placenta is fully formed, the extracellular matrix and vessels of the endometrial stroma are remodeled [7]. How uNK cells and other uterine immune cells reconcile their tissue-specific homeostatic functions with their innate destructive potential is unclear and we have discussed this in another recent review article [8]. Indeed, uNK cells facilitate tissue remodeling, trophoblast invasion, and feto-placental growth, but can also inflame and destroy tissues to defend from pathogens. The potential conflict of interest of tissue immune cells may be resolved by division of labor among subgroups of uNK and ILCs, where some take up roles in homeostasis and others in immunity. For instance, both humans and mice have at least three subsets of uNK cells [1, 2] and in mice, there is some evidence coming from gene expression studies that support this notion. For example, mouse tissue-resident uNK cells may specialize in tissue remodeling, while other uNK cells resembling peripheral NK cells and known as conventional NK cells (cNK) may take up immune defense roles [9]. This review will focus on uNK cells, while we have described more broadly uterine ILCs in other article [10].

### Misnomers and naming names in the extended NK cell family

When discovered in 1975, NK cells were appropriately named after their innate killing instinct [11], but *‘killers become builders during pregnancy’*, was famously suggested in 2006 by Le Bouteiller and Tabiasco [12] commenting on a landmark paper on human dNK cells [13]. Thus, the name we use to refer to the most abundant immune cell population in the uterus is effectively a misnomer. Nevertheless, in an era of immunology when more immune cell subsets are discovered and renamed than we can keep up with [14], it may be wiser to resist the temptation of introducing new names and instead perpetuate the misnaming for the sake of clarity (Table 1). NK cells are the founding member of the ILC family, with the Lymphoid Tissue inducer (LTi) cells discovered some 20 years after and other ILCs from 2008 [15] onward. In this review ‘NK’ refers to all human NK cells (universally CD3^–^CD56^+^, although there is a minor CD56^–^ NK cell subset) regardless of their tissue or species of origin. Within these, ‘pbNK’ specifically indicates human peripheral blood CD3^–^CD56^+^ ILCs, and ‘cNK’ indicates mouse spleen CD3^–^NK1.1^+^CD49b(DX5)^+^ ILCs or cells that phenotypically match them, e.g., cNK are found in mouse uterus and liver. Because human pbNK and mouse cNK cells may be analogous functionally, also human pbNK are sometimes called cNK. Every tissue has its complement of NK cells, including fetal liver, bone marrow, thymus, spleen, lymph nodes, brain, liver, salivary glands, kidney, pancreas, and of course, uterus. By convention the name of NK cells in that tissue is modified by including the first letter of the name of the tissue, hence ‘uNK’ indicates either human CD45^+^Lin^–^CD56^+^ ILCs or mouse CD45^+^Lin^–^NK1.1^+^NKp46^+^ ILCs found in cell suspensions isolated upon digestion of uterine tissue (Table 2). This tissue comes from either human voluntary pregnancy termination at 6–12 weeks of pregnancy or dams culled at various gestational days (gd) and mostly around mid-gestation (gd9.5), mouse pregnancy being 19–21 days, depending on laboratory strains. Because human uNK cells are located in the mucosa, they are often referred to as decidual NK cells (dNK). In mice, uNK cells are also found in the myometrium, where they also specifically accumulate in a gestation-specific structure called mesometrial lymphoid aggregate of pregnancy (MLaP), which is embedded between the two layers of smooth muscle, away from the invading trophoblast and surrounding the uterine artery branch that shoots through the uterus wall. Because they are found both in the decidua and in the myometrium, mouse uterine NK cells are more often referred to as uNK cells rather than dNK cells. Accordingly, we will use dNK specifically for human cells, while uNK may indicate cells of both species. Endometrial (eNK) cells are intended as human or mouse uterine NK cells extracted from the non-pregnant endometrium [16]. Human uNK cells can be collected longitudinally and non-invasively from the menstrual blood [17, 18]. These cells may be informative of their tissue of origin, although one ought to be aware that they have been exposed to physiological tissue breakdown prior to collection.

**Table 1:** NK cells in human (Hu) and mouse (Mo)

Name	Phenotype	Description
NK	Hu CD3^–^ CD56^+^ and Mo CD3^–^ NK1.1^+^	ILC family member, found in blood, lymphoid tissues, and many organs
pbNK	Hu CD3^–^ CD56^+^ and Mo CD3^–^ NK1.1^+^ CD49b(DX5)^+^	Peripheral blood NK cells, including CD56^dim^CD16^+^ killers and CD56^bright^CD16^−^ cytokine producers
cNK	Hu CD3^–^ CD56^+^ and Mo CD3^–^NK1.1^+^ CD49b(DX5)^+^	Conventional human CD56^dim^CD16^+^ or mouse CD3^-^NK1.1^+^DX5^+^ NK cells in blood or any tissue, including spleen, uterus, and liver
dNK	Hu CD45^+^ Lin^–^ CD56^+++^	NK cells in human decidua
uNK	Hu CD45^+^ Lin^–^ CD56^+++^ and Mo CD45^+^ Lin^–^ NK1.1^+^ NKp46^+^	NK cells in human decidua (70% of all lymphocytes) and in mouse decidua or myometrium (30% of all lymphocytes)
eNK	Hu CD45^+^ Lin^–^ CD56^+++^	Endometrial NK cells

**Table 2:** uNK cell subsets in the pregnant human (Hu) or mouse (Mo) uterus^*^

Name	Phenotype	Description
dNK1	Hu CD45^+^ Lin^–^ CD56^+++^ CD49a^+^ KIR^hi^ NKG2A^hi^ LILRB1^hi^ CD39^hi^	NK cells in human decidua (55%)
dNK2	Hu CD45^+^ Lin^–^ CD56^+++^ CD49a^+^ KIR^lo^ NKG2A^hi^ CD103^lo^	NK cells in human decidua (15%)
dNK3/ieILC1	Hu CD45^+^Lin^–^CD56^+++^ CD49a^+^ CD69^hi^ CD103^hi^ NKG2D^hi^ CD161^hi^ KIR^lo^ NKG2A^+/–^ NKp44^+/–^	NK cells in human decidua (15%)
dNKp	Hu CD45^+^ Lin^–^CD56^+++^ CD69^lo^ Ki-67^hi^ NKG2A^hi^ KIR^+/–^	Proliferating human dNK cells (5%)
trNK	Mo CD45^+^ Lin^–^ NK1.1^+^ NKp46^+^ Tbet^+^ Eomes^+^ CD49a^+^	Mouse tissue-resident uNK cells (50%)
cNK	Mo CD45^+^ Lin^–^ NK1.1^+^ NKp46^+^ Tbet^+^ Eomes^+^ CD49b(DX5)^+^	Mouse conventional NK cells in the uterus (40%)
uILC1	Mo CD45^+^ Lin^–^ NK1.1^+^ NKp46^+^ Tbet^+^ Eomes^–^ CD49a^+^	Eomes^–^ mouse tissue-resident uNK cells that resemble ILC1 in other organs, e.g., the liver (10%)

*For more details on human dNK see ref. [10] and for mouse uNK see ref. [2]. Proportions and phenotypes of uNK cells and their subsets vary throughout the menstrual cycle and in pregnancy in humans [21] as well as with puberty, pregnancy, and breastfeeding in the mouse [9].

### Uterine NK cells

NK cells represent the main member of the ILC family [19]. Five main human CD45^+^Lin^–^ CD56^+^ dILC subsets are currently identified: decidual NK cells (dNK1, dNK2, and dNK3), ILC3s, and proliferating NK cells (dNKp) [20]. In dILCs, dNK1 accounts for the most abundant subset (55%), followed by dNK2 (~15%), and dNK3 (~15%). dNK1 express receptors including Killer-cell immunoglobulin-like receptors (KIR), CD94/NKG2A, and LILRB1, indicating that they particularly respond to HLA class I ligands, including maternal HLA-A, -B, -C, and -E or fetal HLA-C, -E or -G, which are expressed by extravillous trophoblasts (EVT). Following stimulation, dNK2 and dNK3 secrete more cytokines and chemokines than dNK1, including GM-CSF and XCL1, which can act on EVTs. The relationship of these human dILCs to those in the mouse uterus is still unknown. Three murine CD45^+^Lin^–^ NK1.1^+^NKp46^+^ uILC subsets are currently identified: Eomes^+^CD49a^+^ tissue-resident NKs (trNK), Eomes^–^ CD49a^+^ ILC1s (also tissue-resident), and Eomes^+^CD49a^–^ cNK [9]. Abundant in early pregnancy, trNKs express factors that promote arterial transformation. cNKs expand after placental development and secrete IFN-γ, while uILC1s expand in subsequent pregnancies and express CXCR6, a memory marker in other lymphocytes. Based on cell surface phenotype, mouse uterine trNK cells share markers with human dNK1, whereas mouse uterine cNK resemble contaminant CD16^+^ pbNK found in the human uterus.

**Fig. 1:**
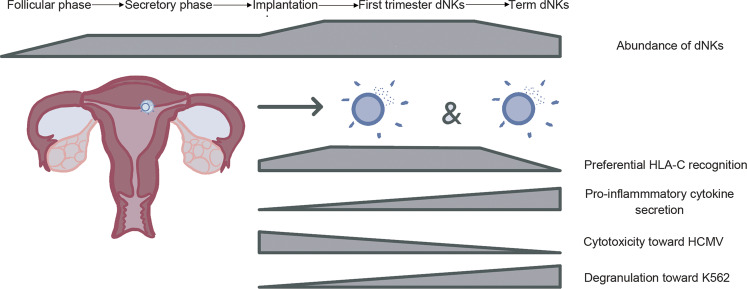
dNKs through the reproductive timeline.

Proportions and phenotypes of uNK cells and their subsets vary throughout the menstrual cycle and in pregnancy in humans [21] as well as with puberty, pregnancy, and breastfeeding in the mouse [9]. How these shifts are related to other dynamic changes in the uterus during pregnancy is unclear. In humans, dNK cells peak in frequency (~70% of the total lymphocytes) during the first trimester and diminish in numbers midway through gestation. Only small numbers of dNKs are present at term [22]. Compared to the first-trimester dNKs, term dNKs show increased degranulation toward K562 and upon PMA/ionomycin stimulation, reduced cytotoxicity towards HCMV-infected cells, reduced HLA-C recognition, and distinct protein and gene expression profiles (Fig. 1). In mice, the proportion of total uNK cells in the uterus is the highest (~30% of the total lymphocytes) at mid-gestation and, within uNK cells, trNK cells peak at gd5.5 to then decrease at mid-gestation, when cNK cells increase. Like human dNK, also mouse trNK and cNK cells decline during late gestation [9, 23].

dNKs are functionally and phenotypically unique compared to other tissue NK cells or pbNKs. They display a distinctive morphology with large granules, secrete a specific set of chemokines, and express a unique combination of cell surface markers [20]. Unlike pbNKs, dNKs are poorly cytotoxic as they are unable to polarize granules to the immune synapse [22]. The poor killing activity of dNKs is surprising given the size of the granules (about triple the size of pbNK granules) [20], and their content, with abundant perforin and granzyme B [24, 25]. Interestingly, poorly cytotoxic pbNK cells of immunodeficient patients affected by the Chediak-Higashi syndrome share some features with dNK cells [26–29]. Rather than triggering cytotoxicity, the interactions between dNK and EVT may activate the release of soluble factors that promote maternal spiral artery transformation, which increases blood, O_2_, and nutrients supply for the growing fetus [30]. Further phenotypic differences between dNK and pbNK include upregulated CD57, CD16, and DNAM-1 and downregulated NKG2A on pbNK cells, which correlate with increased KIR co-expression [31, 32]. In contrast, increased KIR co-expression on dNK correlates with upregulated LILRB1, Ki-67, NKp30, NKG2A, and downregulated NKG2D, CD161, and T-bet [20]. 2B4 is co-activating in pbNKs, but inhibitory in dNKs [33], and LILRB1 is inhibitory in pbNKs, but may activate dNKs [34]. These differences illustrate the wide range of phenotypic and functional features of dNK cells during pregnancy and due to the specific uterine microenvironment. Deep phenotyping of eNK cells [35] and comparisons between cells of women with a history of healthy or complicated pregnancies may provide new biomarkers to predict pregnancy disorders.

### Development of uterine NK cells

We have discussed the current understanding of uNK cell development in another review [10] and summarize the main points here. While it is established that NK cells originate from bone marrow precursors, one unanswered key question is whether uNK cells derive from circulating CD34^+^ hematopoietic progenitor cells (HPCs) or NK lineage-committed precursors that migrate to the uterus. Following bone marrow transfer, NK cells are detected in the uterus of both humans and mice, suggesting that they can arise from transferred HPCs [36, 37]. Human NK-committed precursors are found in blood and can differentiate into mature NK cells in the response to IL-15 [38]. However, CD34^+^ progenitors are also found in the human uterus and can differentiate into NK cells in vitro [39]. Therefore, bone-marrow-derived NK precursors and stem cells resident in the uterus can both contribute to uNK populations. Indeed CD56^+^ NK cells develop in the human uterus xenografted into immunodeficient mice, in response to steroid hormones [40]. Recent data from HLA-mismatched uterus transplants show that uNK can derive from the circulating cells [21]. Human pbNK cells also acquire features of uNK cells when exposed to certain cytokines, including transforming growth factor-β (TGF-β), suggesting that peripheral NK cells also can contribute to uNK populations [41]. Parabiosis studies in mice suggest that tissue-resident uNK cells proliferate during early pregnancy, while recruitment of circulating NK cells increases uNK cell numbers later in gestation [42].

## Homeostasis

### Interactions of uNK cells with decidual stroma cells (dSC)

Decidual stromal cells (dSC) regulate the unique immune microenvironment of the decidua. This is brilliantly illustrated by how mouse dSC epigenetically silence *CXCL9* and *CXCL10*, thus limiting T-cell recruitment to the decidua [43]. In a recent single-cell RNA-sequencing analysis of the maternal-fetal interface, three subsets of human stromal cells have been described: dS1, dS2, and dS3 [1]. dS1 and dS2 cells express *IGF-1* and *IL-15*, respectively [44, 45], suggesting that dS2 may promote the development of dNK cells from CD34^+^ hematopoietic precursors present in human decidua [39]. Producing interleukin-15 (IL-15) [46] and TGF-β1 [41], dSC can significantly modulate the uterine immune microenvironment. In vitro, TGF-β, IL-15, and IL-18 convert pbNK cells into dNK-like cells by inducing the expression of CD9, CD49a, CD103, CXCR3, and CXCR4 [41, 47], suggesting the possibility that at least part of dNK cells originate from peripheral NK cells, [21]. In addition to influencing the phenotype of NK cells and macrophages, dSC may also influence dNK function by downregulating both cytotoxicity and secretion of IFN-γ, mainly through the immunosuppressive action of TGF-β [48]. Secretion of IFN-γ is a point of divergence between human and mouse uNK cells because human uNK do not procude much IFN-γ while mouse uNK cells do secrete it physiologically and actually IFN-γ is the key cytokine driving the necessary vascular changes in murine pregnancy [49]. Within mouse uNK subsets, cNK cells are the main IFN-γ producers, while trNK cells mainly produce homeostatic and vaso-active factors [9, 50]. Nevertheless, human dNK cells can be induced to produce IFN-γ upon culture in vitro [13] and a population of pregnancy-trained dNK cells displays epigenetic modification that eases translation of the *IFNG* gene [18]. Producing chemokines CXCL10/IP-10, CXCL11, and CXCL12 /SDF-1, dSC attract NK cells by binding to receptors CXCR3 [51] and CXCR4 expressed by pbNK and dNK3 [52]. dS2 and dS3 cells express *LGALS9* and *CLEC2D*, which bind TIM3 and KLRB1 on dNK subsets. dS3 cells also express *CXCL14* [1], which stimulates the migration of NK cells binding to unknown receptors [53].

dSC may also contribute to dNK cell education, a process driven by the interaction of inhibitory NK receptors with self MHC molecules. While the specific biological significance of NK cell education remains largely unknown, a comparison of dNK and pbNK inhibitory NK repertoire and self-MHC reveals that NK education operates differentially across tissues [54]. HLA-B dimorphism at position -21 of the leader peptide encodes either methionine (M) or threonine (T) which lead, respectively, to high or low HLA-E expression, resulting in either strong (-21 M/M or M/T) or weak (-21 T/T) NKG2A educators [55]. In women who are genetically programmed to favor NKG2A ligation and education, NKG2A was recently found as protective in pregnancy as it is required for appropriate maternal vascular adaptation, fetal growth, and normal placental gene expression  [56]. Aberrant placentation upon loss of NKG2A was confirmed by transcriptomic studies revealing inadequate vasculature transformation and perturbed gene expression pathways [56, 57]. Independent of maturation status, NKG2A^+^ dNKs are more diverse and functionally responsive than NKG2A^–^counterparts [55, 56]. Finally, NKG2A KO dams carried fetuses affected by asymmetric fetal growth restriction (FGR). Taken together, these data demonstrate that NKG2A education influences pregnancy outcomes.

### Interactions of uNK cells with extravillous trophoblast (EVT)

Trophoblasts are specialized cells differentiating from fetal extraembryonic tissues. They are indeed the first cells that differentiate from the zygote, eventually forming the placenta [58]. Essentially, human trophoblast can be divided into syncytiotrophoblast, which forms the outer layer, and cytotrophoblast on the inner side. Cytotrophoblast is further divided into villous trophoblast, which forms the villi, and extravillous trophoblast (EVT), which is made of cells that bud off the villi to replace maternal endothelial cells or invade deep into the decidua and the myometrium. Trophoblast invasion is shallower in mice, where giant trophoblast cells are loosely analogous to human EVT. First-trimester human EVT cells have a unique human leukocyte antigen (HLA) repertoire, expressing the classical class I molecule HLA-C, and non-classical HLA-E and HLA-G, whereas HLA-A and HLA-B, and class II molecules HLA-DP, HLA-DQ, and HLA-DR are absent. This unique MHC composition enables EVT to modulate subsets of uNK cells, which express combinations of cognate receptors for HLA-C (KIR2DL1, KIR2DS1, KIR2DL2, KIR2DL3, KIR2DS4, and KIR2DS5), HLA-E (CD94/NKG2A and CD94/NKG2C), and HLA-G (KIR2DL4 and LILRB1). Uterine arteries remodeling and placentation are thought to be regulated by these interactions. HLA-C expression is increased on human uterine stromal cells at the onset of pregnancy. The KIR repertoire in dNKs is different from endometrial eNK and pbNK cells, and the expression of HLA-C binding KIRs on dNK increases during the first trimester [59]. HLA-C allotypes can be one of two groups depending on a single amino acid residue on position 80 of the heavy chain: C1^+^HLA-C allotypes have Lys while C2^+^HLA-C allotypes have Asn [60]. A certain combination of maternal KIR and fetal HLA-C are associated with pre-eclampsia and birth weight—immunogenetics of pregnancy complications has been reviewed previously [60, 61]. Women with HLA-C2 have decreased expression of the corresponding receptor, KIR2DL1, while women with HLA-C1 have increased expression of the corresponding receptor, KIR2DL3 [54]. Interestingly, maternal HLA-C has no significant influence on the expression of KIR2DL1 or KIR2DL3 in pbNK cells [54]. These findings suggest that the mechanisms that determine the repertoire of dNK and the effect of self-MHC on NK education may differ in uNK cells compared with pbNK. HLA-E serves as the ligand of the inhibitory receptor CD94/NKG2A and its activating counterpart CD94/NKG2C. HLA-G interacts with KIR2DL4 and LILRB1 [62]. In particular, HLA-G is a dimeric form only expressed by the EVT and its derived peptides allow greater HLA-E expression, thus influencing binding strength to CD94/NKG2A. Additionally, HLA-G can trigger the secretion of cytokines, proangiogenic factors, and growth factors from dNK cells by interacting with KIR2DL4 [63]. Specific dNKs can acquire HLA-G from EVT and display it on their surface via trogocytosis, which is postulated to prolong intracellular signaling for an undefined physiologic purpose [64]. In dNK subsets, dNK1 cells show the greatest expression of KIRs (KIR2DS1, KIR2DS4, KIR2DL1, KIR2DL2, and KIR2DL3), CD94/NKG2A, and LILRB1 compared with dNK2 and dNK3, suggesting that it is dNK1 cells that particularly interact with EVT. Specifically, dNKs regulate the depth of trophoblast invasion and facilitate spiral artery transformation via the release of cytokines including GM-CSF (granulocyte-macrophage colony-stimulating factor), XCL1 (lymphotactin), and pro-angiogenic factors, which directly act on blood vessels [13, 25, 65]. These findings are confirmed in vitro, as dNK demonstrate enhanced functions upon KIR2DS1 or KIR2DS4 cross-linking than upon stimulation with by K562 targets or PMA and ionomycin, which are good activators of pbNK cell functions [13, 25, 65].

Besides the receptor−ligand interactions, the regulation of trophoblast on uNK cells involves a complex network of secreted factors. Upon its establishment at the implantation site, trophoblast can modulate uNK migration, survival, proliferation, phenotype, and function by secreting a range of cytokines and chemokines such as TGF-β, IL-6, CXCL8/ IL-8, CXCL12/ SDF1, and CCL2/MCP1. IL-6 and CXCL8 secreted by EVTs can induce CCL14 and CXCL6 expression in endothelial cells. This crosstalk promotes dNK cells and decidual macrophages infiltration to the early remodeling decidual spiral arteriole wall [66].

### Cytokines

Implantation and EVT invasion are accompanied by modulations of molecular mediators of inflammation, such as prostaglandin E2 (PGE_2_), Tumor necrosis factor (TNF), IL-6, IL-1β, leukemia inhibitory factor (LIF), and IFN-γ [67]. However, the absence of neutrophil infiltration, which is the cellular hallmark of the acute inflammatory response, clearly shows that the response of the decidualized endometrium partially overlaps but does not fully recapitulate the stereotypical inflammatory response that follows tissue injury. In mice, IFN-γ produced by uNK cells is the key factor to remodel the spiral arteries. PGE2 signaling increases vascular permeability and activates NK cells, macrophages, and other regulatory immune cells [68]. TNF synthesized by NK cells, T cells, macrophages, and DCs, and by decidual stromal cells, trophoblast cells, and placenta can induce MMP-9 production in uNK cells, which indirectly affects maternal vascular remodeling [69]. TNF plays an important role in embryo implantation, placentation, and pregnancy progression [70]. However, increased TNF is associated with miscarriage, fetal loss, pre-eclampsia, preterm birth, and endometriosis [71]. TNF inhibitors are readily available for the treatment of autoimmune conditions and have been used in assisted reproductive technology [72]. TGF-β1 plays a significant role in embryo implantation, decidual angiogenesis, and EVT invasion [73]. TGF-β1 in the uterus is produced predominately by Tregs, DCs, and epithelial cells [68, 73]. Both humans and murine uNK cells express the receptor for TGF-β. TGF-β1 suppresses the expression of activating NK cell receptors such as NKG2D and NKp30, but increases CD56 expression in human dNK and pbNK cells [74, 75]. Furthermore, TGF-β1 can downregulate IFNG/ VEGF expression in human dNK cells [75] and convert pbNK cells into dNK cells in vitro [41].

### Metabolites

Pregnancy involves metabolic adaptations. Concentrations of bioactive molecules such as steroid hormones and micronutrients change throughout gestation [76]. In human plasma samples, steroid hormones including estriol-16-glucuronide, estrone 3-sulfate, and progesterone increase over 50% during pregnancy, while most lipids or lipid-like molecules, such as monoacylglycerides, and lyso-phosphatidylcholines decrease. Most of these metabolites originate from the adrenal cortex and gonads, and return to baseline promptly postpartum [76]. The dS3 subset of decidual stromal cells expresses genes such as *CYP11A1* involved in steroid biosynthesis [1]. Hormones are critically involved in a successful pregnancy, and hormonal imbalance can lead to serious pregnancy complications.

During early pregnancy, uNK cells accumulate and expand significantly at the fetal–maternal interface, and this accumulation may be related to the hormone-rich mucosal microenvironment [77]. Human uNK cells express estrogen receptor (ER)β1, ER beta cx/beta 2, and glucocorticoid receptor (GR), but not the estrogen receptor α or progesterone receptors [78, 79]. The distribution of murine uNK cells during early pregnancy is regulated by estrogen and progesterone, and their effects can be abolished by specific antagonism of their nuclear receptors [80]. Estrogen, especially estradiol (E2), can directly promote uNK migration and secretion of (C-C motif) ligand 2 (CCL2) to promotes vascular remodeling [81]. Progesterone promotes human uterine stromal cells and endothelial cells secreting chemokines, including CXCL8/IL-8, CXCL10/IP-10, CXCL12/SDF-1, CX3CL1/Fractalkine, and CCL2/MCP-1, which can recruit pbNK, dNK, and T cells to the uterus during early pregnancy [52]. Besides, progesterone can stimulate human endometrial stromal cells secreting IL-15 to promote uNK self-renewal [82]. Progesterone reduces CD69 and IFN-γ expression induced by CpG and IL-12 in human uNK cells and mouse splenic NK cells [79]. Some of the immunological effects of progesterone are mediated by the progesterone-induced blocking factor (PIBF). PIBF serum concentrations increase throughout pregnancy and decrease before labor [83]. During spontaneous miscarriage or preterm delivery, urinary and serum PIBF concentrations are lower than normal [84]. In mice, anti-PIBF monoclonal antibody treatment or anti-progesterone treatment in the peri-implantation period result in decreased PIBF^+^ dNK cells number and increased splenic NK activity, together with impaired implantation and increased resorption rates [85]. These data suggest that the pregnancy-protective effect of the progesterone and PIBF may be due to their inhibitory activity on NK cells. The glucocorticoid prednisolone is reported to reduce dNK cell concentrations in recurrent miscarriage and in patients who suffer from repeated implantation failure and may have with high uNK cell concentrations; however, small sample-size reports did not reveal a significant beneficial effect of this treatment on pregnancy outcomes [86, 87]. Endogenous glucocorticoids induce PD-1 expression on spleen NK cells and reduce IFN-γ production in mice upon viral infection [88], although both human and murine NK cells display minimal PD-1 expression [89]. Human chorionic gonadotrophin (hCG) is one of the first hormones synthesized by the embryo and has immunomodulatory properties during pregnancy [90]. hCG acts on many immune cells including uNK, regulatory T (Treg) cells, B cells, DCs, macrophages, and monocytes [90, 91]. uNKs do not express luteinizing hormone/choriogonadotropin receptor, which is the classical hCG receptor expressed by the endometrial surface. Instead, hCG regulates the proliferation of uNKs through another receptor, the mannose receptor (CD206) [92].

Besides hormonal changes, significant changes in glucose, lipids, proteins, vitamins, and calcium metabolism also occur during pregnancy [93]. At present, the effects of these changes on uNK cells are unclear, although there is information available on their effect on pbNK cells. For example, levels of vitamin D increase significantly in maternal serum during pregnancy and vitamin D is metabolized further into an active form of vitamin D, 1,25-dihydroxyvitamin D_3_ (1,25(OH)_2_D_3_). In addition to calcium homeostasis and bone metabolism, 1,25(OH)_2_D_3_ exerts an immunomodulatory effect on multiple immune cells including NK cells, T cells, B cells, and macrophages in pregnancy [94]. 1,25(OH)_2_D_3_ reduces pbNK-cell cytotoxicity and cytokine secretion in women with recurrent pregnancy loss [95]. Vitamin D_3_ upregulated protein 1 (VDUP1) is required for the development of mouse NK cells [96]. Vitamin D_3_ may have an effect also on human dNK cells as dNK cells express the vitamin D receptor [97].

### Hypoxia

Hypoxia is a physiological feature of the uterus in early pregnancy. Rodesch et al. [98] recorded that the endometrial average oxygen tension (PO_2_) in the human endometrium was around 39.6 mm Hg (5% O_2_) in pregnancies at 8–10 week’s gestation [98]. The PO_2_ in the endometrium and placenta rises steeply between 10 and 12 weeks [99]. Cells use hypoxia-inducible factors (HIFs) to sense, adapt, and respond to low oxygen. The stability and function of transcription factor HIFα proteins (HIF-1α, HIF-2α, and HIF-3α isoforms) are post-translationally regulated by hydroxylation. Uterine expression of HIF-1α and HIF-2α is increased and up-regulated by progesterone, and estrogen in early pregnant mice [100]. HIFα regulates placental angiogenesis [101], luminal epithelium detachment, and trophoblast cell differentiation and invasion [102–104].

Local tissue oxygen tension performs as one of the key physiological regulatory mechanisms for immune responses. In murine spleen and lymph nodes, the level of exposure to hypoxia was high in NK cells than in NKT, T, and B cells [105], indicating NK cells may have a stronger ability to adapt to low oxygen environment. Hypoxia may contribute to the phenotypic and functional adaption of uNK to the uterus. In contrast to pbNK cells, uNK cells have low cytotoxic ability, and secrete pro-angiogenic factors. Hypoxia in the presence of TGF-β1 (1% O_2_) decreases cytotoxicity and enhances VEGF-α production by human dNK cells [106]. Hypoxia also impairs human pbNK cell cytotoxicity, cytokine and chemokine secretion, and the expression of activating NK cell receptors [107, 108]. Depletion of *Hif1a* in mouse NK cells or short-term hypoxia does not affect NK cell maturation or receptor repertoire. Similarly, splenic NK cell degranulation and IFN-γ expression are not affected under hypoxia, when stimulated with ligands, cytokines, or PMA/ionomycin; however, these functions were decreased in response to certain tumor cell lines [109]. Genetic or pharmacological ablation of Hif1a in NK cells mouse and human NK cells results in elevated cytotoxicity and IFN-γ expression in response to tumor cells [108].

Hypoxia improves lymphoid differentiation potentials of lymphoid-primed multipotent progenitors (by HIF-1α) and pro-T/ NK cells (by HIF-2α) in vitro [110]. The hypoxic nature of the uterine microenvironment raises the possibility that uNK cells are derived in situ  because hypoxia contributes to phenotypically and functionally converting pbNK cells to uNK cells. Indeed, a combination of hypoxia, TGF-β1, and demethylating agents converts human pbNK cells to uNK-like cells [106], suggesting that uNK may also derive from pbNK cells. Genes that are typically upregulated in response to hypoxia (such as *Ang* and *Hif3a*) were upregulated in mouse uILCs at gd9.5, compared with liver ILC, indicating that uterine hypoxia microenvironment may affect uILCs [9].

Overall, hypoxia may contribute to the phenotypic and functional adaption of uNK cells to the uterus, and meanwhile, uNK cells regulate vascular remodeling and subsequently modulate oxygen delivery and trophoblast lineage decisions [111]. The hypoxia affecting NK cell function might be dependent on conditions in the microenvironment. For example, prolonged exposure to hypoxia and other factors, such as inflammatory cytokines, might drive HIFs stabilization and its functions [108].

The uterus must manage the stress of constant tissue reorganization, cell division, and, upon implantation, hypoxia, vascular remodeling, and trophoblast invasion. Peripheral NK cells can sense stress during infection and cancer, for example through NKG2D that binds stress-induced ligands (e.g., MICA, MICB, ULBP1-3, MULT-1, or in mice, Rae-1). Whether uNK cells also sense stress through NKG2D or other receptors is unknown. In pregnant mice, NKG2D binds Rae-1 expressed on trophoblast and triggers IFN-γ production by uNK cells [112]. In pregnant rats, lack of uNK cells affects trophoblast lineage decision with consequent deeper trophoblast invasion in response to hypoxia [102]. In non-pregnant women who are pharmacologically treated to stop excessive bleeding through progesterone blocking, the endometrial arteries display thick walls and reduced vessel lumens. The endometrial vascular phenotype of anti-progesterone-treated women is similar to that found in the decidua of NK-deficient pregnant mice. Interestingly, anti-progesterone treatment interferes with IL-15 production by endometrial stromal cells, leading to uNK cell deficiency in women [113]. Therefore, uNK cells are responsible for arterial remodeling both in the non-pregnant endometriam and in the decidua.

### First pregnancy and beyond

In humans, first-time pregnant women (nulliparous) are exposed to a greater risk of poor placentation and placental development than women experiencing repeated pregnancies [114]. A subset of human NKG2C^high^ dNK cells, which seem to “remember” pregnancy, may facilitate subsequent pregnancies by enhanced IFN-γ and VEGF production [18]. Transcriptome and epigenetic analysis revealed that these “pregnancy-trained” dNK cells are indeed a unique and tissue-specific population [18]. Pregnancy-trained human dNK cells most closely resemble dNK1 cells with higher expression of NKG2C, LILRB1, and KIR [1, 18]. ‘Priming’ of dNK1 subsets during the first pregnancy may make them respond more effectively in subsequent pregnancies [1]. Murine dILC1 expressing CXCR6 expand in second pregnancies indicating they may act as memory cells [9]. Cytokines IL-12, IL-15, and IL-18 and NK cell receptors CD16, CD2, and NKG2C can induce memory-like pbNK cells [115, 116]. Epigenetic imprinting and metabolomic programming of memory-like pbNK cells occur in chronic human CMV infection [117]. It is unknown whether pregnancy-trained uNK cells are relatable to cytokines- or CMV-induced memory-like pbNK cells; however, specific receptor−ligand interactions, inflammatory cytokines, and hypoxia in the unique uterine microenvironment may well contribute to the generation of memory-like cells among uNK subsets.

## Biology of disease

A careful synergy of physiological signaling is required to guide proper implantation, intrauterine development, and balancing of fetal−maternal immune interactions for successful placentation and pregnancy [118]. Disruption of these tightly regulated processes can result in life-threatening complications for mom and baby [119]. In this section, we briefly summarize potential uNK cell roles in pregnancy complications.

### Great obstetrical syndromes

Defective deep placentation due to inadequate spiral artery transformation is found in a host of pregnancy complications termed ‘Great Obstetrical Syndromes’ (GOS). Syndromic in nature, GOS are frustratingly complex to predict and prevent due to their long preclinical period, multiple aetiologies, and interactions between maternal and fetal genomes along with the environment [120]. Specifically, preeclampsia (PE), FGR, preterm labor, preterm premature rupture of membranes, recurrent spontaneous abortion (RSA), and abruption placentae are all GOS responsible for maternal and perinatal morbidity [121]. All GOS are thought to originate from defective EVT invasion causing insufficient arterial remodeling, with consequent disruptively high blood flow speed but reduced gas and nutrient exchanges, resulting in placental stress and damage.

**Fig. 2:**
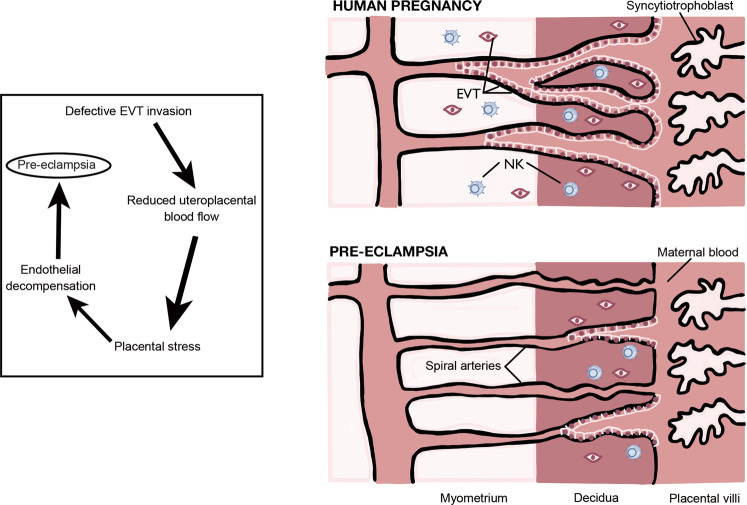
The pathophysiology of Pre-Eclampsia.

Although the exact pathophysiology mechanism underlying the GOS is undefined, the immune system and uNK cells may be part of it. Figure 2 illustrates the main events leading to pre-eclampsia. Pre-eclampsia refers to the onset of hypertension and proteinuria after 20 weeks of gestation, occurring in 2–8% of the pregnancies as a consequence of abnormal placentation and subsequent maternal vascular dysfunction. Progression to the eclamptic phase can be fatal and requires immediate delivery of the placenta, and therefore the baby, to resolve [122]. PE and FGR are intimately connected as high resistance vessel blood flow and marked pro-inflammatory secretion in the uterine microenvironment found as a consequence of PE often cause FGR. Infants born with a birth weight below the 10th percentile termed small for gestational age (SGA), can be pathological due to chromosomal abnormalities, intrauterine infections, or FGR. Inadequate spiral artery remodeling from defective placentation is the primary etiology of FGR, in which forced elevation in umbilical artery pressure cause placental endothelial decompensation from blood spurting and increased fetal middle cerebral artery blood flow (a phenomenon known as brain sparing). Ultimately, FGR manifests as clinically recognized asymmetric head to abdominal circumference and has been associated with behavioral/cognitive consequences [123].

**Fig. 3:**
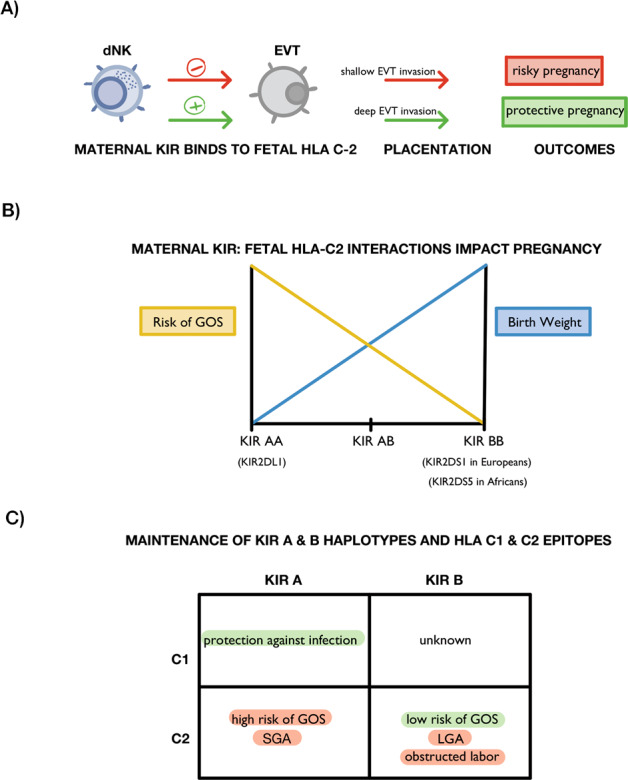
KIR on maternal dNKs and HLA-C on fetal EVT affect pregnancy outcomes.

The immunogenetics of PE and other pregnancy complications is reviewed elsewhere [60, 61] and summarized in Fig. 3. Changes in the uterine microenvironment have been described in pregnancy complications. Compared with normal pregnancy subjects, Tregs are decreased both in placental bed and the circulation in women with pre-elampsia [124], while pro-inflammatory Th17 cells and cytokines IFN-γ and IL-4 are increased [125]. Additionally, enhanced activation of M1 macrophages occurs [126]. dNK cells can regulate other immune cells, including induction of Tregs and suppressing Th17 cells [127]. In PE, the phenotypes, cytotoxicity, and production of cytokines and angiogenic factors by NK cells are changed [128]. For example, proposed as a potential diagnostic tool, women with PE display an attenuation of the relative proportion of CD56^bright^/NKp46^+^ pbNKs and elevation of CD56^+^/NKp44^+^ pbNKs, in which the former persists from the onset of PE until delivery [128]. Despite these reported associations, pbNKs are not representative of the NK cells at the fetal-maternal interface and therefore pbNK findings cannot be straightforwardly extrapolated to uNKs [127, 129, 130]. Reported associations of uNK cell decline or elevation with PE and FGR are debatable [131–135]; however inappropriate uNK activity seems to be associated with the incidence of PE and FGR [65].

Increased number of cytotoxic NK cells in PE patients has also been reported [127]. Women with PE were also found to display elevated expression of perforin, and granzyme B compared on both pbNKs and uNKs [135, 136]. Interestingly, these molecules are markedly more elevated in early rather than late-onset PE women [135]. In PE mothers, overly cytotoxic uNKs are postulated to target first-trimester trophoblast for apoptosis and inhibit their spiral artery invasion and migration [135]. Mimicking of PE via a reduced uterine perfusion pressure (RUPP) model revealed improved FGR and inflammation upon depletion of NKs from the uterine microenvironment [137, 138]. These results suggest that cytolytic uNKs contribute to hypertension, FGR, and inflammation in response to placental ischemia [138].

VEGF-C, a pro-angiogenic factor produced by uNKs, is less expressed in women with PE and FGR [139]. Fu and colleagues suggest that growth-promoting factors osteopontin, pleitrophin, and osteoglycin, which are secreted by CD49a^+^ Eomes^+^ uNKs in both humans and mice, are implicated in FGR prevention. Adoptively transferred, these cells re-established an appropriate uterine microenvironment and reversed impaired fetal growth in the *Nfil3*^*−/−*^ mouse model of FGR [63]. In line with these findings, CD49a^+^ PBX1^+^ uNKs, which promote pleiotrophin and osteoglycin transcription in humans and mice were recently found to be required for optimal fetal growth as reduced or complete loss of PBX1 expression is associated with FGR and RSA [140]. Furthermore, TNF modulatory agents, such as Etanercept, may play a role as a potential therapeutic for PE as TNF-α blockade ameliorated hypertension and reduced mitochondrial ROS and cytolytic NK counts in a RUPP rat model [141]. IL-17 is a candidate mediator of PE as IL-17 blockade in a RUPP model improves the placental and fetal weight, placental VEGF expression. IL-17 blockade also attenuates TNF-α, MIP-3α, and placental cNK cytolytic activity and counts [142]. Emerging evidence from cord blood samples in PE mothers reveals fetal NKs are also dysregulated as there is a higher ratio of effector to regulatory pbNKs [143, 144]. Some reports show loss of uNKs in PE pregnancies complicated with FGR, while only a small uNK decline is seen in PE and normal pregnancies [131]. Genetic inactivation of PI3K p110δ—a crucial molecule for intracellular signaling in leukocytes—causes reduced uterine arterial remodeling associated with lower uNK cell production of IFN-γ, which subsequently lead to FGR in a mouse model [145]. Incidence of PE is also associated with variants of endoplasmic reticulum aminopeptidase 1 and 2 (ERAP1/2) [146], receptors present in the placenta involved in the regulation of blood pressure, angiogenesis,  immune recognition [147], modulation of NK cell and CD8^+^ T responses [148, 149], as well as recruitment of cytotoxic pbNK cells [146]. Perhaps the steady circulation of these cytotoxic pbNKs contributes to the clinical manifestation of PE via dysregulating the innate immune response and aggravating the uterine microenvironment.

RSA and recurrent implantation failure (RIF) are largely idiopathic pregnancy disorders that are distressing and pose serious threats to the mother and fetus. The causes of RSA and RIF are complicated, including chromosomal abnormalities, uterine structure abnormalities, endocrine causes, and infections. The role of immune cells in RSA and RIF is under scrutiny. For example, uNK cells may indirectly ‘sense’ embryos by targeting senescent decidual cells. Medium-conditioned embryos that fail to implant, but not successful embryos, inhibit uNK-cell-mediated killing of senescent decidual cells [150]. This suggests that uNK cells contribute to the selection of healthy embryos. Interestingly, pbNK cells also have the ability to detect senescent cancer cells through receptors NKG2D and DNAM-1 [151]. RSA and RIF are associated with an imbalanced and possibly inflamed uterine immune microenvironment. However, the main obstacle to determine the cellular and molecular mechanism leading to RSA and RIF is that investigations are inevitably conducted after the pathology has occurred and therefore distinguishing causes from consequences is challenging. Nevertheless, comparative scRNA-sequence analysis of pathological and normal utero-placental tissues may shed further light on the potential mechanisms [3].

Stillbirth is the loss of a fetus *in utero*. The definite cause of stillbirth remains elusive; however, besides its association with PE due to placental vascular dysplasia [152], there is evidence indicative of NK cell involvement. Expression of RCAS1, a type II membrane protein expressed in the uterine endometrium and EVTs known to contribute to maternal immune tolerance, was found to be low in placental tissue of women following stillbirth [153]. Also, scaffolding protein Grb2-associated binding protein 3 (Gab3) was recently discovered to be critical for uNK cell expansion, spiral artery remodeling, and appropriate limiting of trophoblast invasion in mice [154]. Notably, Gab3 murine knockout resulted in maternal hemorrhage and stillbirth.

### Infections

How maternal uNK cells and other innate and adaptive immune cells control infection while promoting fetal development is not fully understood. We have reviewed the role of NK cells in infection during pregnancy elsewhere [8]. There is evidence that uNK and other immune cells prevent the spreading of pathogens to the fetus. For example, dNKs display an elegant and unique mechanism to control *Listeria monocytogenes* through the antimicrobial peptide granulysin. Granulysin, stored in intracellular granules, can transfer to infected EVTs via nanotubes to selectively kill intracellular Listeria, without killing the infected EVTs [155]. Here we take human cytomegalovirus (HCMV) as an example to summarize the interactions between intrauterine infection and uNK cells during pregnancy.

HCMV is the most common cause of congenital virus infection, passing from the maternal bloodstream through the placenta to infect the fetus and leading long-term consequences. Around 1 out of 200 babies is born with CMV and about 20% of congenital cases result in serious neurodevelopmental sequelae including hearing and vision loss, seizures, motor delay, intellectual disability, and microcephaly [156]. HCMV infection is also associated with IUGR and placental dysfunction [157]. HCMV infections during pregnancy add to the high occurrence of miscarriage, pre-eclampsia, and preterm birth [158, 159]. Currently, no HCMV vaccines are licensed for clinical use, no reliable prenatal markers for congenital disease are identified, and no prenatal antiviral treatments are available [118].

HCMV infection induces specific changes in the uterine environment during pregnancy. HCMV replicates consistently in decidual stromal cells and epithelial cells of endometrial glands, which results in inflammation and impaired function, and soluble molecule secretion [160]. HCMV can spread from the decidua to chorionic villi in early gestation and attenuate trophoblast invasion and differentiation [157]. Congenital viral infection of the placenta leads to pathological changes, including the development of avascular villi, edema, calcification, hypoxia, and inflammation, and thus causes deleterious effects including placental insufficiency, stillbirth [158], and IUGR [157]. Infections trigger a series of immune microenvironment changes such as activation of the complement system, innate and adaptive immune cells, and secretion of pro-inflammatory cytokines. HCMV-infection modulates uNK cell receptor repertoire and secretory profile [160]. CD56, KIR2DL1, KIR2DL4, and ILT2 decrease while NKp44, NKG2C increase in dNK cells, which also acquire CD16. HCMV-infection also modulates cytokine and chemokine secretion by dNK cells, and increases Granzyme B, which may contribute to eliminating HCMV by cytotoxicity [160]. Acute HCMV infection triggers selective expansion of CD57^+^ NKG2C^hi^ pbNK populations, affecting the expression of their inhibitory receptor [161]. However, this expansion has not been observed in uNK cells.

uNKs accumulate at the site of HCMV-infected tissue and co-localize with infected cells [160, 162]. uNK expressing the activating KIR2DS1 receptor display increased cytotoxicity toward HCMV-infected DSCs, especially when DSCs express the cognate HLA-C2 ligand. KIR2DS1 expression in uNK is associated with greater intracellular content of perforin, granzyme B, and granulysin [20]. Lysis of HCMV-infected dSC by uNKs could limit viral spread to the fetoplacental unit [163]. While pbNKs can degranulate toward HCMV-infected EVT in vitro, it remains uncertain whether they can flux into the decidua in vivo. Interestingly, even if uNKs can kill HCMV-infected DSCs, EVT are more resistant to NK cell cytotoxicity, which could be due to HLA-G expression on EVT [164]. EVT may possess an independent HCMV defense mechanism, such as miRNA or cell-surface death receptor expression. HCMV targets HLA-C for downregulation in order to limit trophoblast invasion, evade viral peptide presentation on maternal T cells [165], and reduce activation of both HLA-C restricted T cells and KIR2DS1^+^ uNK [166].

### Endometriosis and endometrial cancer

Endometriosis is a condition of unknown etiology and unclear pathogenesis that may affect up to  one in ten women in reproductive age with pain, morbidity, and, in some cases, infertility. Several studies have reported that reduced NK cell activity is associated with endometriosis [167]. Recent studies have attempted to assess the correlation of certain combinations of KIR and HLA-C genes with endometriosis [168]. It is reasonable to postulate that reduced NK cell activity may facilitate the extra endometrial growth, which underlies endometriosis, but the mechanisms are unclear. In a case-control study of a small number of patients and controls, the percentage of peritoneal NK cells expressing the inhibitory receptor CD94/NKG2A + NK cells was higher in patients with endometriosis [169]. This suggests a role for CD94/NKG2A and its cognate ligand HLA-E in suppressing NK cell activity in these patients.

Endometrial cancer (EC) is the most common gynecological cancer and the fourth most common malignancy in females. Parity may be negatively associated with EC risk [170], suggesting that modulation of estrogen and progesterone levels affect susceptibility. The presence of CD8^+^ cytotoxic T cells within the tumor microenvironment and loss of HLA class I expression correlates positively and negatively, respectively, with disease outcomes and survival of patients with EC. The presence of NK cells also associates with better outcomes when HLA-E is upregulated, while maintenance of normal HLA-E expression associates with the worst outcomes. HLA-E has a greater affinity for the inhibitory NKG2A rather than the activating NKG2C receptor on NK cells. One possibility to explain the beneficial effect of high HLA-E expression and NK cell is that an increase in HLA-E may saturate NKG2A, thus shifting the interaction toward NKG2C and away from NK inhibition to promote tumor lysis [171].

## Recent developments

Interest in the topic of this review is growing rapidly. Access to unique human samples and data, availability of new technology, including multiple “-omics”, and new mouse models are helping to expand our knowledge of the uterine microenvironment and its NK cells. Using a combination of mouse models, micro-CT scans, and micro-dopplers in mice, we have recently shown that the conserved CD94/NKG2A inhibitory receptor is required to educate mouse uNK cells. Indeed, with a functional Qa1-NKG2A pathway, the uterine micro-environment educates uNK cells effectively to mediate the necessary vascular changes in the uterus that ultimately help fetuses to grow symmetrically and sustain normal brain development [56]. In the same study, we showed that the NKG2A pathway is linked to pre-eclampsia in a meta-GWAS analysis of over 150,000 human pregnancies [56].

Using single-cell profiling, it was recently shown that the decidual micro-environment of patients with recurrent pregnancy loss (RPL) is different from that of normal control pregnancies. One of these differences is that a subset of uNK cells that specializes in supporting fetal growth is reduced in patients. Despite the inevitable complications of comparing a pathological sample with a healthy one, this study helps deciphering the decidual microenvironment with the view of improving diagnosis and therapy of RPL [172].

Also using a combination of mouse models and human endometrial samples, plus uterine tissue of either women who had undergone uterine transplant or monozygotic twins, it was shown recently that endometrial NK cell differentiation is, at least in part, a genetically determined process driven by IL-15. This process happens continuously and in response to both monthly endometrial regeneration and pregnancy [21].

The importance of certain variants of immune system genes *HLA-C* and *KIR* in creating a uterine micro-environment conducive to healthy fetal growth was confirmed recently in an elegant humanized mouse model. Fetal growth was reduced in pups of transgenic females that express a specific variant of the strongly inhibitory human KIR2DL1 receptor on all their NK cells, but only when sires were males expressing the cognate human HLA-C ligand [173]. This supports a working model in which too much inhibition of uNK cells leads to poor fetal growth [174], a model based on human genetic epidemiology evidence [60].

## Concluding remarks

From menarche to menopause the uterus remodels its mucosa every month in response to hormones. During the course of pregnancy and upon blastocyst implantation, the uterus undergoes even more dramatic changes including trophoblast invasion, and vascular remodeling, culminating in the formation of the placenta. These adaptations are accompanied by changes in tissue-resident immune cell populations, hypoxia, and metabolic adaptations. The unique microenvironment of the uterus during pregnancy remodels and adapts the phenotype and function of uNK cells. At the same time, uNK cells promote arterial integrity, decidualization, EVTs invasion, spiral arterial remodeling, early placental formation, and fetal growth. Determining the mechanisms underlying these adaptations will help understand the causes of major pregnancy complications.

## References

[1] Vento-tormo R, Efremova M, Botting RA, Turco MY, Vento-Tormo M, Meyer KB (2018). Single-cell reconstruction of the early maternal–fetal interface in humans. Nature.

[2] Doisne J-M, Balmas E, Boulenouar S, Gaynor LM, Kieckbusch J, Gardner L (2015). Composition, development, and function of uterine innate lymphoid cells. J. Immunol..

[3] Suryawanshi H, Morozov P, Straus A, Sahasrabudhe N, Max K, Garzia A (2018). A single-cell survey of the human first-trimester placenta and decidua. Sci Adv..

[4] Weisblum Y, Panet A, Zakay-Rones Z, Haimov-Kochman R, Goldman-Wohl D, Ariel I (2011). Modeling of human cytomegalovirus maternal-fetal transmission in a novel decidual organ culture. J Virol..

[5] Jauniaux E, Jurkovic D (2012). Placenta accreta: pathogenesis of a 20th century iatrogenic uterine disease. Placenta.

[6] Sojka DK (2020). Uterine natural killer cell heterogeneity: lessons from mouse models. Front Immunol..

[7] Yang F, Zheng Q, Jin L (2019). Dynamic function and composition changes of immune cells during normal and pathological pregnancy at the maternal-fetal interface. Front Immunol..

[8] Shmeleva, E & Colucci, F. Maternal natural killer cells at the intersection between reproduction and mucosal immunity. Mucosal Immunol. 2020. 10.17863/CAM.60546. 10.1038/s41385-020-00374-3 PMC8071844 33903735

[9] Filipovic I, Chiossone L, Vacca P, Hamilton RS, Ingegnere T, Doisne JM (2018). Molecular definition of group 1 innate lymphoid cells in the mouse uterus. Nat Commun..

[10] Huhn O, Zhao X, Esposito L, Moffett A, Colucci F, Sharkey AM (2021). How do uterine natural killer and innate lymphoid cells contribute to successful pregnancy?. Front Immunol..

[11] Kiessling R, Klein E, Wigzell H (1975). ‘Natural’ killer cells in the mouse. I. Cytotoxic cells with specificity for mouse Moloney leukemia cells. Specificity and distribution according to genotype. Eur J Immunol..

[12] Le Bouteiller P, Tabiasco J (2006). Killers become builders during pregnancy. Nat Med.

[13] Hanna J, Goldman-Wohl D, Hamani Y, Avraham I, Greenfield C, Natanson-Yaron S (2006). Decidual NK cells regulate key developmental processes at the human fetal-maternal interface. Nat Med..

[14] Vivier E, Artis D, Colonna M, Diefenbach A, Di Santo JP, Eberl G (2018). Innate lymphoid cells: 10 Years on. Cell.

[15] Cella M, Fuchs A, Vermi W, Facchetti F, Otero K, Lennerz JK (2009). A human natural killer cell subset provides an innate source of IL-22 for mucosal immunity. Nature.

[16] Manaster I, Mizrahi S, Goldman-Wohl D, Sela HY, Stern-Ginossar N, Lankry D (2008). Endometrial NK cells are special immature cells that await pregnancy. J Immunol..

[17] Ivarsson MA, Stiglund N, Marquardt N, Westgren M, Gidlöf S, Björkström NK (2017). Composition and dynamics of the uterine NK cell KIR repertoire in menstrual blood. Mucosal Immunol..

[18] Gamliel M, Goldman-Wohl D, Isaacson B, Gur C, Stein N, Yamin R (2018). Trained memory of human uterine NK cells enhances their function in subsequent pregnancies. Immunity.

[19] Spits H, Bernink JH, Lanier L (2016). NK cells and type 1 innate lymphoid cells: partners in host defense. Nat Immunol..

[20] Huhn O, Ivarsson MA, Gardner L, Hollinshead M, Stinchcombe JC, Chen P (2020). Distinctive phenotypes and functions of innate lymphoid cells in human decidua during early pregnancy. Nat Commun..

[21] Strunz B, Bister J, Jönsson H, Filipovic I, Crona-Guterstam Y, Kvedaraite E (2021). Continuous human uterine NK cell differentiation in response to endometrial regeneration and pregnancy. Sci Immunol..

[22] de Mendonça Vieira R, Meagher A, Crespo ÂC, Kshirsagar SK, Iyer V, Norwitz ER (2020). Human term pregnancy decidual NK cells generate distinct cytotoxic responses. J Immunol..

[23] Sojka DK, Yang L, Yokoyama WM (2019). Uterine natural killer cells. Front Immunol..

[24] King A, Wooding P, Gardner L, Loke YW (1993). Immunology: expression of perforin, granzyme A and TIA-1 by human uterine CD56+ NK cells implies they are activated and capable of effector functions. Hum Reprod..

[25] Koopman LA, Kopcow HD, Rybalov B, Boyson JE, Orange JS, Schatz F (2003). Human decidual natural killer cells are a unique NK cell subset with immunomodulatory potential. J Exp Med..

[26] Roder JC, Haliotis T, Klein M, Korec S, Jett JR, Ortaldo J (1980). A new immunodeficiency disorder in humans involving NK cells. Nature.

[27] Chiang SCC, Wood SM, Tesi B, Akar HH, Al-Herz W, Ammann S (2017). Differences in granule morphology yet equally impaired exocytosis among cytotoxic T cells and NK cells from Chediak–Higashi syndrome patients. Front Immunol..

[28] Gil-Krzewska A, Wood SM, Murakami Y, Nguyen V, Chiang S, Cullinane AR (2016). Chediak-Higashi syndrome: lysosomal trafficking regulator domains regulate exocytosis of lytic granules but not cytokine secretion by natural killer cells. J. Allergy Clin Immunol..

[29] Dominovic M, Laskarin G, Glavan Gacanin L, Haller H, Rukavina D (2016). Colocalization of granulysin protein forms with perforin and LAMP-1 in decidual lymphocytes during early pregnancy. Am J Reprod Immunol..

[30] Gaynor LM, Colucci F (2017). Uterine natural killer cells: functional distinctions and influence on pregnancy in humans and mice. Front Immunol..

[31] Björkström NK, Riese P, Heuts F, Andersson S, Fauriat C, Ivarsson MA (2010). Expression patterns of NKG2A, KIR, and CD57 define a process of CD56dim NK-cell differentiation uncoupled from NK-cell education. Blood.

[32] Wang F, Zhou Y, Fu B, Wu Y, Zhang R, Sun R (2014). Molecular signatures and transcriptional regulatory networks of human immature decidual NK and mature peripheral NK cells. Eur J Immunol..

[33] Vacca P, Pietra G, Falco M, Romeo E, Bottino C, Bellora F (2006). Analysis of natural killer cells isolated from human decidua: evidence that 2B4 (CD244) functions as an inhibitory receptor and blocks NK-cell function. Blood.

[34] Li C, Houser BL, Nicotra ML, Strominger JL (2009). HLA-G homodimer-induced cytokine secretion through HLA-G receptors on human decidual macrophages and natural killer cells. Proc Natl Acad Sci USA..

[35] Feyaerts D, Kuret T, van Cranenbroek B, van der Zeeuw-Hingrez S, van der Heijden O, van der Meer A (2018). Endometrial natural killer (NK) cells reveal a tissue-specific receptor repertoire. Hum Reprod..

[36] Lysiak JJ, Lala PK (1992). In situ localization and characterization of bone marrow-derived cells in the decidua of normal murine pregnancy. Biol Reprod..

[37] Taylor HS (2004). Endometrial cells derived from donor stem cells in bone marrow transplant recipients. JAMA.

[38] Male V, Hughes T, McClory S, Colucci F, Caligiuri MA, Moffett A (2010). Immature NK cells, capable of producing IL-22, are present in human uterine mucosa. J Immunol..

[39] Vacca P, Vitale C, Montaldo E, Conte R, Cantoni C, Fulcheri E (2011). CD34+ hematopoietic precursors are present in human decidua and differentiate into natural killer cells upon interaction with stromal cells. Proc Natl Acad Sci USA..

[40] Matsuura-Sawada R, Murakami T, Ozawa Y, Nabeshima H, Akahira J, Sato Y (2005). Reproduction of menstrual changes in transplanted human endometrial tissue in immunodeficient mice. Hum Reprod..

[41] Keskin DB, Allan DS, Rybalov B, Andzelm MM, Stern JN, Kopcow HD (2007). TGFbeta promotes conversion of CD16+ peripheral blood NK cells into CD16- NK cells with similarities to decidual NK cells. Proc Natl Acad Sci USA..

[42] Sojka DK, Yang L, Plougastel-Douglas B, Higuchi DA, Croy BA, Yokoyama WM (2018). Cutting edge: local proliferation of uterine tissue-resident NK cells during decidualization in mice. J Immunol..

[43] Nancy P, Tagliani E, Tay CS, Asp P, Levy DE, Erlebacher A (2012). Chemokine gene silencing in decidual stromal cells limits T cell access to the maternal-fetal interface. Science.

[44] Schumacher A, Sharkey DJ, Robertson SA, Zenclussen AC (2018). Immune cells at the fetomaternal interface: how the microenvironment modulates immune cells to foster fetal development. J Immunol..

[45] Ni F, Sun R, Fu B, Wang F, Guo C, Tian Z (2013). IGF-1 promotes the development and cytotoxic activity of human NK cells. Nat Commun..

[46] Kitaya K, Yasuda J, Yagi I, Tada Y, Fushiki S, Honjo H (2000). IL-15 expression at human endometrium and decidua. Biol Reprod..

[47] Levy C, Siewiera J, Gouilly J, Hocine H (2015). Natural cytotoxicity receptor splice variants orchestrate the distinct functions of human natural killer cell subtypes. Nat Commun..

[48] Ashkar AA, Black GP, Wei Q, He H, Liang L, Head JR (2003). Assessment of requirements for IL-15 and IFN regulatory factors in uterine NK cell differentiation and function during pregnancy. J Immunol..

[49] Ashkar BAA, Santo JP, Di, Croy BA (2000). Interferon γ contributes to initiation of uterine vascular modification, decidual integrity, and uterine natural killer cell maturation during normal murine pregnancy. J Exp Med..

[50] Chen Z, Zhang J, Hatta K, Lima PD, Yadi H, Colucci F (2012). DBA-lectin reactivity defines mouse uterine natural killer cell subsets with biased gene expression. Biol Reprod..

[51] Sentman CL, Meadows SK, Wira CR, Eriksson M (2004). Recruitment of uterine NK cells: induction of CXC chemokine ligands 10 and 11 in human endometrium by estradiol and progesterone. J Immunol..

[52] Carlino C, Stabile H, Morrone S, Bulla R, Soriani A, Agostinis C (2008). Recruitment of circulating NK cells through decidual tissues: a possible mechanism controlling NK cell accumulation in the uterus during early pregnancy. Blood.

[53] Starnes T, Rasila KK, Robertson MJ, Brahmi Z, Dahl R, Christopherson K (2006). The chemokine CXCL14 (BRAK) stimulates activated NK cell migration: implications for the downregulation of CXCL14 in malignancy. Exp Hematol..

[54] Sharkey AM, Xiong S, Kennedy PR, Gardner L, Farrell LE, Chazara O (2015). Tissue-specific education of decidual NK cells. J Immunol..

[55] Horowitz A, Djaoud Z, Nemat-Gorgani N, Blokhuis J, Hilton HG, Béziat V (2016). Class I HLA haplotypes form two schools that educate NK cells in different ways. Sci Immunol..

[56] Shreeve N, Depierreux D, Hawkes D, Traherne JA, Sovio U, Huhn O (2021). The CD94/NKG2A inhibitory receptor educates uterine NK cells to optimize pregnancy outcomes in humans and mice. Immunity.

[57] Hung T-H, Skepper JN, Charnock-Jones DS, Burton GJ (2002). Hypoxia-reoxygenation: a potent inducer of apoptotic changes in the human placenta and possible etiological factor in preeclampsia. Circ Res..

[58] Turco MY, Moffett A (2019). Development of the human placenta. Development.

[59] Male V, Sharkey A, Masters L, Kennedy PR, Farrell LE, Moffett A (2011). The effect of pregnancy on the uterine NK cell KIR repertoire. Eur J Immunol..

[60] Moffett A, Colucci F (2015). Co-evolution of NK receptors and HLA ligands in humans is drivenby reproduction. Immunol Rev..

[61] Colucci F (2017). The role of KIR and HLA interactions in pregnancy complications. Immunogenetics.

[62] Xu X, Zhou Y, Wei H (2020). Roles of HLA-G in the maternal-fetal immune microenvironment. Front Immunol..

[63] Fu B, Zhou Y, Ni X, Tong X, Xu X, Dong Z (2017). Natural killer cells promote fetal development through the secretion of growth-promoting factors. Immunity.

[64] Tilburgs T, Evans JH, Crespo ÂC, Strominger JL (2015). The HLA-G cycle provides for both NK tolerance and immunity at the maternal-fetal interface. Proc Natl Acad Sci USA..

[65] Xiong S, Sharkey AM, Kennedy PR, Gardner L, Farrell LE, Chazara O (2013). Maternal uterine NK cell-activating receptor KIR2DS1 enhances placentation. J Clin Invest..

[66] Choudhury RH, Dunk CE, Lye SJ, Aplin JD, Harris LK, Jones RL (2017). Extravillous trophoblast and endothelial cell crosstalk mediates leukocyte infiltration to the early remodeling decidual spiral arteriole wall. J Immunol..

[67] Griffith OW, Chavan AR, Protopapas S, Maziarz J, Romero R (2017). Embryo implantation evolved from an ancestral inflammatory attachment reaction. Proc Natl Acad Sci USA..

[68] Ghaebi M, Nouri M, Ghasemzadeh A, Farzadi L (2017). Immune regulatory network in successful pregnancy and reproductive failures. Biomed Pharmacother..

[69] Naruse K, Lash GE, Innes BA, Otun HA, Searle RF, Robson SC (2009). Localization of matrix metalloproteinase (MMP)-2, MMP-9 and tissue inhibitors for MMPs (TIMPs) in uterine natural killer cells in early human pregnancy. Hum Reprod..

[70] Yuan J, Li J, Huang S-Y, Sun X (2015). Characterization of the subsets of human NKT-like cells and the expression of Th1/Th2 cytokines in patients with unexplained recurrent spontaneous abortion. J Reprod Immunol..

[71] Alijotas-reig J, Esteve-valverde E (2017). Tumor necrosis factor-alpha and pregnancy: focus on biologics. an updated and comprehensive review. Clin Rev Allergy Immunol..

[72] Moffett A, Shreeve N (2015). First do no harm: uterine natural killer (NK) cells in assisted reproduction. Hum Reprod..

[73] Plaks V, Birnberg T, Berkutzki T, Sela S, BenYashar A, Kalchenko V (2008). Uterine DCs are crucial for decidua formation during embryo implantation in mice. J Clin Invest..

[74] Allan DS, Rybalov B, Awong G, Zúñiga-Pflücker JC, Kopcow HD, Carlyle JR (2010). TGF-b affects development and differentiation of human natural killer cell subsets. Eur J Immunol.

[75] Zhang J, Dunk CE, Shynlova O, Caniggia I, Lye SJ (2019). TGFb1 suppresses the activation of distinct dNK subpopulations in preeclampsia. EBioMedicine.

[76] Liang L, Rasmussen MH, Piening B, Shen X, Chen S, Röst H (2020). Metabolic dynamics and prediction of gestational age and time to delivery in pregnant women. Cell.

[77] Moffett-King A (2002). Natural killer cells and pregnancy. Nat Rev Immunol..

[78] Henderson TA, Saunders PTK, Moffett-King A, Groome NP, Critchley HOD (2003). Steroid receptor expression in uterine natural killer cells. J Clin Endocrinol Metab..

[79] Guo W, Li P, Zhao G, Fan H, Hu Y, Hou Y (2012). Glucocorticoid receptor mediates the effect of progesterone on uterine natural killer cells. Am J Reprod Immunol..

[80] Kuang H, Peng H, Xu H, Zhang B, Peng J, Tan Y (2010). Hormonal regulation of uterine natural killer cells in mouse preimplantation uterus. J Mol Histol..

[81] Gibson DA, Greaves E, Critchley HOD, Saunders PTK (2015). Estrogen-dependent regulation of human uterine natural killer cells promotes vascular remodelling via secretion of CCL2. Hum Reprod.

[82] Gong H, Chen Y, Xu J, Xie X, Yu D, Yang B (2017). The regulation of ovary and conceptus on the uterine natural killer cells during early pregnancy. Reprod Biol Endocrinol..

[83] Polgár B, Nagy E, Mikó É, Varga P, Szekeres-Barthó J (2004). Urinary progesterone-induced blocking factor concentration is related to pregnancy outcome. Biol Reprod..

[84] Hudić I, Szekeres-Bartho J, Stray-Pedersen B, Fatušić Z, Polgar B, Ećim-Zlojutro V (2016). Lower urinary and serum progesterone-induced blocking factor in women with preterm birth. J Reprod Immunol..

[85] Csabai T, Pallinger E, Kovacs AF, Miko E, Bognar Z, Szekeres-Bartho J (2020). Altered immune response and implantation failure in progesterone-induced blocking factor-deficient mice. Front Immunol..

[86] Cooper S, Laird SM, Mariee N, Li TC, Metwally M (2019). The effect of prednisolone on endometrial uterine NK cell concentrations and pregnancy outcome in women with reproductive failure. A retrospective cohort study. J Reprod Immunol..

[87] Tang AW, Alfirevic Z, Turner MA, Drury JA, Small R, Quenby S (2013). A feasibility trial of screening women with idiopathic recurrent miscarriage for high uterine natural killer cell density and randomizing to prednisolone or placebo when pregnant. Hum Reprod..

[88] Quatrini L, Wieduwild E, Escaliere B, Filtjens J, Chasson L, Laprie C (2018). Endogenous glucocorticoids control host resistance to viral infection through the tissue-specific regulation of PD-1 expression on NK cells. Nat Immunol..

[89] Judge SJ, Dunai C, Aguilar EG, Vick SC, Sturgill IR, Khuat LT (2020). Minimal PD-1 expression in mouse and human NK cells under diverse conditions. J Clin Invest..

[90] Gridelet V, Perrier d'Hauterive S, Polese B, Foidart JM, Nisolle M, Geenen V (2020). Human chorionic gonadotrophin: new pleiotropic functions for an “old” hormone during pregnancy. Front Immunol..

[91] Schumacher A, Costa SD, Zenclussen AC (2014). Endocrine factors modulating immune responses in pregnancy. Front Immunol..

[92] Kane N, Kelly R, Saunders PTK, Critchley HOD (2009). Proliferation of uterine natural killer cells is induced by human chorionic gonadotropin and mediated via the mannose receptor. Endocrinology.

[93] Soma-pillay P, Nelson-piercy C, Tolppanen H, Mebazaa A (2016). Physiological changes in pregnancy. Cardiovasc J Afr.

[94] Cyprian F, Lefkou E, Varoudi K, Girardi G (2019). Immunomodulatory effects of vitamin D in pregnancy and beyond. Front Immunol..

[95] Ota K, Dambaeva S, Kim MW, Han AR, Fukui A, Gilman-Sachs A (2015). 1,25-Dihydroxy-vitamin D3 regulates NK-cell cytotoxicity, cytokine secretion, and degranulation in women with recurrent pregnancy losses. Eur J Immunol..

[96] Lee KN, Kang HS, Jeon JH, Kim EM, Yoon SR, Song H (2005). VDUP1 is required for the development of natural killer cells. Immunity.

[97] Tamblyn JA, Jeffery LE, Susarla R, Lissauer DM, Coort SL, Garcia AM (2019). Transcriptomic analysis of Vitamin D responses in uterine and peripheral NK cells. Reproduction.

[98] Rodesch F, Simon P, Donner C, Jauniaux E (1992). Oxygen measurements in endometrial and trophoblastic tissues during early pregnancy. Obstet Gynecol..

[99] Jauniaux E, Watson AL, Hempstock J, Bao YP, Skepper JN, Burton GJ (2000). Onset of maternal arterial blood flow and placental oxidative stress: a possible factor in human early pregnancy failure. Am J Pathol..

[100] Daikoku T, Matsumoto H, Gupta RA, Das SK, Gassmann M, DuBois RN (2003). Expression of hypoxia-inducible factors in the peri-implantation mouse uterus is regulated in a cell-specific and ovarian steroid hormone-dependent manner. Evidence for differential function of HIFs during early pregnancy. J Biol Chem..

[101] Befani C, Liakos P (2018). The role of hypoxia-inducible factor-2 alpha in angiogenesis. J Cell Physiol..

[102] Chakraborty D, Karim Rumi MA, Konno T, Soares MJ (2011). Natural killer cells direct hemochorial placentation by regulating hypoxia-inducible factor dependent trophoblast lineage decisions. Proc Natl Acad Sci USA..

[103] Cowden Dahl KD, Fryer BH, Mack FA, Compernolle V, Maltepe E, Adelman DM (2005). Hypoxia-inducible factors 1 and 2 regulate trophoblast differentiation. Mol Cell Biol..

[104] Matsumoto L, Hirota Y, Saito-Fujita T, Takeda N, Tanaka T, Hiraoka T (2018). HIF2a in the uterine stroma permits embryo invasion and luminal epithelium detachment. J Clin Invest..

[105] Ohta A, Diwanji R, Kini R, Subramanian M, Ohta A, Sitkovsky M (2011). In vivo T cell activation in lymphoid tissues is inhibited in the oxygen-poor microenvironment. Front Immunol..

[106] Cerdeira AS, Rajakumar A, Royle CM, Lo A, Husain Z, Thadhani RI (2013). Conversion of peripheral blood NK cells to a decidual NK-like phenotype by a cocktail of defined factors. J Immunol..

[107] Parodi M, Raggi F, Cangelosi D, Manzini C, Balsamo M, Blengio F (2018). Hypoxia modifies the transcriptome of human NK cells, modulates their immunoregulatory profile, and influences NK cell subset migration. Front Immunol..

[108] Ni J, Wang X, Stojanovic A, Zhang Q, Wincher M, Bühler L (2020). Single-cell RNA sequencing of tumor-infiltrating NK cells reveals that inhibition of transcription factor HIF-1α unleashes NK cell activity. Immunity.

[109] Krzywinska E, Kantari-Mimoun C, Kerdiles Y, Sobecki M, Isagawa T, Gotthardt D (2017). Loss of HIF-1α in natural killer cells inhibits tumour growth by stimulating non-productive angiogenesis. Nat Commun..

[110] Chabi S, Uzan B, Naguibneva I, Rucci J, Fahy L, Calvo J (2019). Hypoxia regulates lymphoid development of human hematopoietic progenitors. Cell Rep..

[111] Chakraborty D, Rumi MAK, Soares MJ (2012). NK cells, hypoxia, and trophoblast cell differentiation. Cell Cycle.

[112] Carayannopoulos LN, Barks JL, Yokoyama WM, Riley JK (2010). Murine trophoblast cells induce NK cell interferon-gamma production through KLRK1. Biol Reprod..

[113] Wilkens J, Male V, Ghazal P, Forster T, Gibson DA, Williams AR (2013). Uterine NK cells regulate endometrial bleeding in women and are suppressed by the progesterone receptor modulator asoprisnil. J. Immunol..

[114] Yeh C-C, Chao K-C, Huang SJ (2013). Innate immunity, decidual cells, and preeclampsia. Reprod Sci..

[115] Romee R, Schneider SE, Leong JW, Chase JM, Keppel CR, Sullivan RP (2012). Cytokine activation induces human memory-like NK cells. Blood.

[116] Pahl JHW, Cerwenka A, Ni J (2018). Memory-like NK cells: remembering a previous activation by cytokines and NK cell receptors. Front Immunol..

[117] Cichocki F, Wu CY, Zhang B, Felices M, Tesi B, Tuininga K (2018). ARID5B regulates metabolic programming in human adaptive NK cells. J Exp Med..

[118] Weisblum Y, Panet A, Haimov-Kochman R, Wolf DG (2014). Models of vertical cytomegalovirus (CMV) transmission and pathogenesis. Semin Immunopathol..

[119] Mor G, Cardenas I (2010). The immune system in pregnancy: a unique complexity. Am J Reprod Immunol..

[120] Di Renzo GC (2009). The great obstetrical syndromes. J Matern Fetal Neonatal Med.

[121] Brosens I, Pijnenborg R, Vercruysse L, Romero R (2011). The ‘Great Obstetrical Syndromes’ are associated with disorders of deep placentation. Am J Obstet Gynecol..

[122] Sibai B, Dekker G, Kupferminc M (2005). Pre-eclampsia. Lancet.

[123] Sharma D, Shastri S, Sharma P (2016). Intrauterine growth restriction: antenatal and postnatal aspects. Clin Med Insights Pediatr..

[124] Sasaki Y, Darmochwal-Kolarz D, Suzuki D, Sakai M, Ito M, Shima T (2007). Proportion of peripheral blood and decidual CD4+ CD25 bright regulatory T cells in pre-eclampsia. Clin Exp Immunol..

[125] Santner-Nanan B, Peek MJ, Khanam R, Richarts L, Zhu E, Fazekas de St Groth B (2009). Systemic increase in the ratio between Foxp3+ and IL-17-producing CD4 + T cells in healthy pregnancy but not in preeclampsia. J Immunol..

[126] Rambaldi MP, Weiner E, Mecacci F, Bar J, Petraglia F (2019). Immunomodulation and preeclampsia. Best Pract Res Clin Obstet Gynaecol..

[127] Fu B, Li X, Sun R, Tong X, Ling B, Tian Z (2012). Natural killer cells promote immune tolerance by regulating in flammatory TH17 cells at the human maternal–fetal interface. Proc Natl Acad Sci USA..

[128] Fukui A, Yokota M, Funamizu A, Nakamua R, Fukuhara R, Yamada K (2012). Changes of NK cells in preeclampsia. Am J Reprod Immunol..

[129] Saito S (2000). Cytokine network at the feto-maternal interface. J Reprod Immunol..

[130] Moffett A, Regan L, Braude P (2004). Natural killer cells, miscarriage, and infertility. BMJ.

[131] Eide IP, Rolfseng T, Isaksen CV, Mecsei R, Roald B, Lydersen S (2006). Serious foetal growth restriction is associated with reduced proportions of natural killer cells in decidua basalis. Virchows Arch..

[132] Lockwood CJ, Huang SJ, Chen CP, Huang Y, Xu J, Faramarzi S (2013). Decidual cell regulation of natural killer cell-recruiting chemokines: implications for the pathogenesis and prediction of preeclampsia. Am J Pathol..

[133] Stallmach T, Hebisch G, Orban P, Lü X (1999). Aberrant positioning of trophoblast and lymphocytes in the feto-maternal interface with pre-eclampsia. Virchows Arch..

[134] Bachmayer N, Rafik Hamad R, Liszka L, Bremme K, Sverremark-Ekström E (2006). Aberrant uterine natural killer (NK)-cell expression and altered placental and serum levels of the NK-cell promoting cytokine interleukin-12 in pre-eclampsia. Am J Reprod Immunol..

[135] Du, M, Wang W, Huang L, Guan X, Lin W, Yao J, et al. Natural killer cells in the pathogenesis of preeclampsia: a double-edged sword. J Matern Fetal Neonatal Med. 2020. 10.1080/14767058.2020.1740675. 10.1080/14767058.2020.1740675 32188324

[136] Darmochwal-Kolarz D, Rolinski J, Leszczynska-Goarzelak B, Oleszczuk J (2002). The expressions of intracellular cytokines in the lymphocytes of preeclamptic patients. Am J Reprod Immunol..

[137] Elfarra J, Amaral LM, McCalmon M, Scott JD, Cunningham MW, Gnam A (2017). Natural killer cells mediate pathophysiology in response to reduced uterine perfusion pressure. Clin. Sci..

[138] Cornelius DC (2018). Preeclampsia: from inflammation to immunoregulation. Clin Med Insights Blood Disord..

[139] Kalkunte S, Lai Z, Norris WE, Pietras LA, Tewari N, Boij R (2009). Novel approaches for mechanistic understanding and predicting preeclampsia. J Reprod Immunol..

[140] Zhou Y, Fu B, Xu X, Zhang J, Tong X, Wang Y (2020). PBX1 expression in uterine natural killer cells drives fetal growth. Sci Transl Med..

[141] Cunningham MW, Jayaram A, Deer E, Amaral LM, Vaka VR, Ibrahim T (2020). Tumor necrosis factor alpha (TNF-α) blockade improves natural killer cell (NK) activation, hypertension, and mitochondrial oxidative stress in a preclinical rat model of preeclampsia. Hypertens Pregnancy.

[142] Travis OK, White D, Baik C, Giachelli C, Thompson W, Stubbs C (2020). Interleukin-17 signaling mediates cytolytic natural killer cell activation in response to placental ischemia. Am J Physiol Regul Integr Comp Physiol..

[143] Loewendorf AI, Nguyen TA, Yesayan MN, Kahn DA (2015). Preeclampsia is characterized by fetal NK cell activation and a reduction in regulatory T cells. Am J Reprod Immunol..

[144] Bujold E, Chaiworapongsa T, Romero R, Gervasi MT, Espinoza J, Goncalves LF (2003). Neonates born to pre-eclamptic mothers have a higher percentage of natural killer cells (CD3-/CD56+16+) in umbilical cord blood than those without pre-eclampsia. J Matern Fetal Neonatal Med.

[145] Kieckbusch J, Balmas E, Hawkes DA, Colucci F (2015). Disrupted PI3K p110δ signaling dysregulates maternal immune cells and increases fetal mortality in mice. Cell Rep..

[146] Seamon K, Kurlak LO, Warthan M, Stratikos E, Strauss JF, Mistry HD (2020). The differential expression of ERAP1/ERAP2 and immune cell activation in pre-eclampsia. Front Immunol..

[147] Cifaldi L, Romania P, Lorenzi S, Locatelli F, Fruci D (2012). Role of endoplasmic reticulum aminopeptidases in health and disease: from infection to cancer. Int J Mol Sci..

[148] Cifaldi L, Romania P, Falco M, Lorenzi S, Meazza R, Petrini S (2015). ERAP1 regulates natural killer cell function by controlling the engagement of inhibitory receptors. Cancer Res..

[149] Zervoudi E, Saridakis E, Birtley JR, Seregin SS, Reeves E, Kokkala P (2013). Rationally designed inhibitor targeting antigen-trimming aminopeptidases enhances antigen presentation and cytotoxic T-cell responses. Proc Natl Acad Sci USA..

[150] Kong C-S, Ordoñez AA, Turner S, Tremaine T, Muter J, Lucas ES (2021). Embryo biosensing by uterine natural killer cells determines endometrial fate decisions at implantation. FASEB J Publ Fed Am Soc Exp Biol..

[151] Soriani A, Zingoni A, Cerboni C, Iannitto ML, Ricciardi MR, Di Gialleonardo V (2009). ATM-ATR-dependent up-regulation of DNAM-1 and NKG2D ligands on multiple myeloma cells by therapeutic agents results in enhanced NK-cell susceptibility and is associated with a senescent phenotype. Blood.

[152] Yang X, Yang Y, Yuan Y, Liu L, Meng T (2020). The roles of uterine natural killer (NK) cells and KIR/HLA-C combination in the development of preeclampsia: a systematic review. Biomed Res Int..

[153] Wicherek L, Popiela TJ, Galazka K, Dutsch-Wicherek M, Opławski M, Basta A (2005). Metallothionein and RCAS1 expression in comparison to immunological cells activity in endometriosis, endometrial adenocarcinoma, and endometrium according to menstrual cycle changes. Gynecol Oncol..

[154] Sliz, A, Locker K, Lampe K, Godarova A, Plas DR, Janssen EM, et al. Gab3 is required for IL-2- and IL-15-induced NK cell expansion and limits trophoblast invasion during pregnancy. Sci Immunol. 2019;4:eaav3866. 10.1126/sciimmunol.aav3866 PMC7068803 31375526

[155] Crespo ÂC, Mulik S, Dotiwala F, Ansara JA, Sen Santara S, Ingersoll K (2020). Decidual NK cells transfer granulysin to selectively kill bacteria in trophoblasts. Cell.

[156] Slavuljica I, Kveštak D, Huszthy PC, Kosmac K, Britt WJ, Jonjić S (2015). Immunobiology of congenital cytomegalovirus infection of the central nervous system—the murine cytomegalovirus model. Cell Mol Immunol..

[157] Pereira L, Petitt M, Fong A, Tsuge M, Tabata T, Fang-Hoover J (2014). Intrauterine growth restriction caused by underlying congenital cytomegalovirus infection. J Infect Dis..

[158] Iwasenko JM, Howard J, Arbuckle S, Graf N, Hall B, Craig ME (2011). Human cytomegalovirus infection is detected frequently in stillbirths and is associated with fetal thrombotic vasculopathy. J Infect Dis..

[159] Xie F, Hu Y, Magee LA, Money DM, Patrick DM, Krajden M (2010). An association between cytomegalovirus infection and pre-eclampsia: a case-control study and data synthesis. Acta Obstet Gynecol Scand..

[160] Bouteiller P (2013). Human cytomegalovirus infection elicits new decidual natural killer cell effector functions. PLoS Pathog.

[161] Lopez-Vergès S, Milush JM, Schwartz BS, Pando MJ, Jarjoura J, York VA (2011). Expansion of a unique CD57+NKG2C hi natural killer cell subset during acute human cytomegalovirus infection. Proc Natl Acad Sci USA..

[162] Tabata T, Petitt M, Fang-Hoover J, Pereira L (2019). Survey of cellular immune responses to human cytomegalovirus infection in the microenvironment of the uterine–placental interface. Med Microbiol Immunol..

[163] Crespo ÂC, van der Zwan A, Ramalho-Santos J, Strominger JL, Tilburgs T (2017). Cytotoxic potential of decidual NK cells and CD8+ T cells awakened by infections. J Reprod Immunol..

[164] Tilburgs T, Crespo ÂC, van der Zwan A, Rybalov B, Raj T, Stranger B (2015). Human HLA-G+extravillous trophoblasts: immune-activating cells that interact with decidual leukocytes. Proc Natl Acad Sci USA..

[165] Tilburgs T, Strominger JL (2013). CD8+ effector T cells at the fetal-maternal interface, balancing fetal tolerance and antiviral immunity. Am J Reprod Immunol..

[166] Foley B, De Santis D, Lathbury L, Christiansen F, Witt C (2008). KIR2DS1-mediated activation overrides NKG2A-mediated inhibition in HLA-C C2-negative individuals. Int Immunol..

[167] Thiruchelvam U, Wingfield M, O’Farrelly C (2015). Natural killer cells: key players in endometriosis. Am J Reprod Immunol..

[168] Chou Y-C, Chen CH, Chen MJ, Chang CW, Chen PH, Yu MH (2020). Killer cell immunoglobulin-like receptors (KIR) and human leukocyte antigen-C (HLA-C) allorecognition patterns in women with endometriosis. Sci Rep..

[169] Galandrini R, Porpora MG, Stoppacciaro A, Micucci F, Capuano C, Tassi I (2008). Increased frequency of human leukocyte antigen-E inhibitory receptor CD94/NKG2A-expressing peritoneal natural killer cells in patients with endometriosis. Fertil Steril..

[170] Wu Q-J, Li YY, Tu C, Zhu J, Qian KQ, Feng TB (2015). Parity and endometrial cancer risk: a meta-analysis of epidemiological studies. Sci Rep..

[171] Versluis MAC, Marchal S, Plat A, de Bock GH, van Hall T, de Bruyn M (2017). The prognostic benefit of tumour-infiltrating natural killer cells in endometrial cancer is dependent on concurrent overexpression of human leucocyte antigen-E in the tumour microenvironment. Eur J Cancer.

[172] Guo C, Cai P, Jin L, Sha Q, Yu Q, Zhang W (2021). Single-cell profiling of the human decidual immune microenvironment in patients with recurrent pregnancy loss. Cell Discov..

[173] Kaur G, Porter CBM, Ashenberg O, Lee J, Riesenfeld SJ, Hofree M, et al. Parental-fetal interplay of immune genes leads to intrauterine growth restriction. Preprint at Europe PMC 2021. 10.1101/2021.03.26.437292.

[174] Kieckbusch J, Gaynor LM, Moffett A, Colucci F (2014). MHC-dependent inhibition of uterine NK cells impedes fetal growth and decidual vascular remodelling. Nat Commun..

